# Overexpression of microRNA-381-3p ameliorates hypoxia/ischemia-induced neuronal damage and microglial inflammation via regulating the C-C chemokine receptor type 2 /nuclear transcription factor-kappa B axis

**DOI:** 10.1080/21655979.2022.2038448

**Published:** 2022-03-04

**Authors:** Yuanmei Che, Jianglong He, Xiaopeng Li, Daxian Wu, Yi Zhang, Guicai Yuan

**Affiliations:** a Department of Infection, The First Affiliated Hospital of Nanchang University, Nanchang, China; b Department of Infection, The Second Affiliated Hospital of Yichun University, Yichun, China

**Keywords:** Ischemic stroke, neuron, miR-381-3p, CCR2, neuroinflammation

## Abstract

microRNAs, as small endogenous RNAs, influence umpteen sophisticated cellular biological functions regarding neurodegenerative and cerebrovascular diseases. Here, we interrogated miR-381-3p’s influence on BV2 activation and neurotoxicity in ischemic and hypoxic environment. Oxygen-glucose deprivation (OGD) was adopted to induce microglial activation and HT-22 neuron damage. Quantitative polymerase chain reaction (qRT-PCR) was taken to check miR-381-3p expression in OGD-elicited BV2 cells and HT-22 neurons. It transpired that miR-381-3p expression was lowered in BV2 cells and HT-22 cells elicited by OGD. miR-381-3p up-regulation remarkably hampered inflammatory mediator expression in BV2 cells induced by OGD and weakened HT22 neuron apoptosis. *In vivo*, miR-381-3p expression was abated in HI rats’ ischemic lesions, and miR-381-3p up-regulation could ameliorate inflammation and neuron apoptosis in their brain. C-C chemokine receptor type 2 (CCR2) was identified as the downstream target of miR-381-3p, and miR-381-3p suppressed the CCR2/NF-κB pathway to mitigate microglial activation and neurotoxicity. Therefore, we believed that miR-381-3p overexpression exerts anti-inflammation and anti-apoptosis in ischemic brain injury by targeting CCR2

## Graphical abstract

**Figure:**
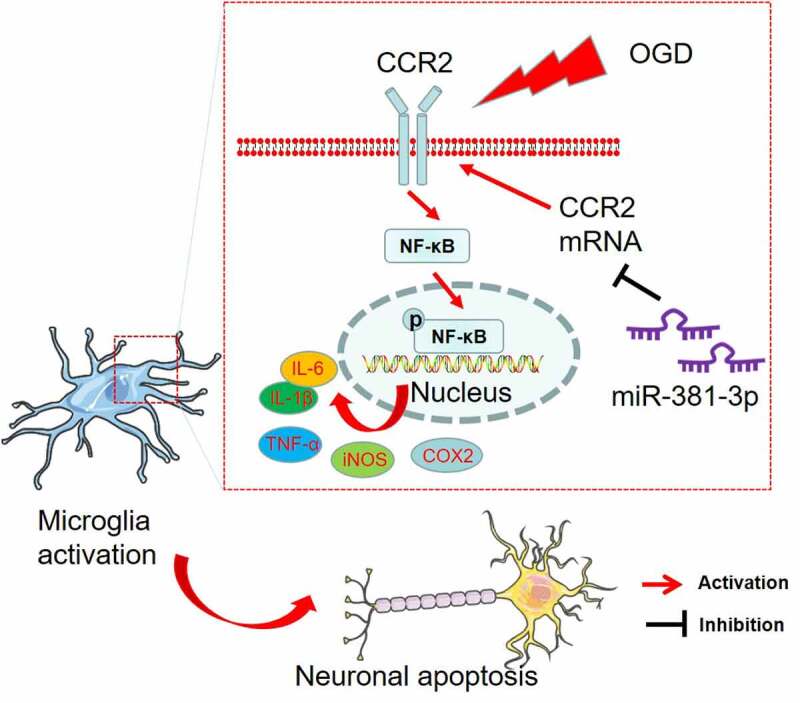

## Introduction

Hypoxic-ischemic brain injury (HIBD), a prevalent brain injury, is the primary underlying pathogenesis of stroke and other neurological diseases. Cerebral hypoxia/ischemia results in neuronal injury/death and ultimately gives rise to severe neurological diseases and even death for patients[1]. Following hypoxic ischemia (HI) damage, microglia will increase inflammation through pro-inflammatory cytokines and chemokines and weaken the functions of mitochondria, thus activating apoptotic pathways and subsequently altering the functions of neurons in the contralateral hemisphere[2]. Prior works have verified that restraint on inflammatory cytokines secreted by microglia can abate inflammation caused by ischemia and hypoxia [3–5]. Notwithstanding, we are still in the dark about the mechanism that modulates microglia activation. Therefore, a deeper understanding of the hypoxic/ischemic process is high on the agenda. We need to search for novel therapies for brain damage arising from hypoxia and ischemia.

microRNAs (miRNAs), with 21–25 nucleotides in length, are a type of highly conserved non-coding RNAs. They, together with complementary sequences in the 3’ -untranslated region, play a significant role in modulating gene expression at the post-transcriptional level through the method of base pairing[6]. miRNAs can influence the progression of tumors as a pro-cancer or anti-cancer gene[7]. Apart from that, recent studies have disclosed that they have become a new therapeutic target for central nervous system (CNS) injury, affecting oxidative stress, inflammation, apoptosis, blood-brain barrier protection, angiogenesis, neurogenesis, and other processes in the central nervous system[8]. For instance, the intraventricular injection of the miR-3473b antagomir into rats preceding middle cerebral artery occlusion (MCAO) considerably attenuates the profiles of cerebral ischemia-induced miR-3473b and pro-inflammatory cytokines and reduces the volume of infarction in rats following MCAO[9]. miR-182-5p is down-regulated in BV2 microglia induced by OGD, but its up-regulation can impede the release of TNF-a, IL-6, and IL-1β in microglia[10]. In the TBI rat model, the profile of miR-711 is uplifted, but miR-711 expression inhibition, a potential therapeutic target for TBI, can weaken the mechanism of apoptosis and reduce neuron death in an Akt-dependent way[11]. Given the studies mentioned above, miRNA can modulate neuroinflammation and neuronal apoptosis in nervous system diseases. miR-381-3p is a member of the miRNA family. In the context of ischemic stroke, overexpression of miR-381-3p may facilitate angiogenesis via Cebpb and Mapk 38 inhibition and suppress inflammatory cytokine release in vascular endothelial cells[12]. Nonetheless, we have little knowledge about how miR-381-3p regulates microglial inflammation in hypoxia/ischemia and how it influences the development of diseases.

Chemokine (C-C motif) receptor 2 (CCR2), representative of the CC chemokine receptors, is extensively distributed in cerebral neurons, astrocytes, and microglia. It is the major chemokine receptor in brain tissues and also the pivotal mediator of neuroinflammation and microglia activation [13,14]. Scores of studies have been made on the influence of CCR2 and its ligands on various inflammatory diseases of the central nervous system, covering multiple sclerosis (MS), Alzheimer’s disease, and ischemic stroke[15]. For instance, in TBI rats, the levels of CCL2 and CCR2 mRNA are considerably elevated. Selective CCR2 antagonist RS504393 can decrease TUNEL staining in the rats and enhance their cognitive functions[16]. In the process of ethanol-triggered microglial activation, a therapy with RS504393 (CCR2 antagonist) prominently attenuates ethanol-caused microglia activation/neuroinflammation and neuronal apoptosis[14]. In light of the above studies, impeding CCR2 signaling axis activation can cramp neuroinflammation development. Moreover, a multitude of miRNAs can prevent inflammation in diseases through targeting CCR2, covering miR-511-3p [17] and miR-155[18]. Nevertheless, the correlation between miR-381-3p and CCR2 in hypoxia/ischemia remains obscure.

The study aims to probe the function of miR-381-3p in modulating microglial polarization and further affecting hippocampal neurogenesis after hypoxia/ischemia. We discovered that miR-381-3p expression was reduced in OGD-elicited BV2 cells and HT-22 cells as well as in the brain tissues of HI rats. miR-381-3p up-regulation could repress inflammatory factors’ expressions in BV2 cells induced by OGD and lessen neuron apoptosis. As evidenced by the online website analysis and dual luciferase activity assay, there was a targeted correlation between miR-381-3p and CCR2, which were negatively associated with each other. Therefore, we conjectured that miR-381-3p mitigate microglial activation and neurotoxicity by targeting CCR2/NF-κB pathway. What we have found in the research may foster an underlying strategy for hypoxic-ischemic encephalopathy.

## Materials and methods

### Cell culture

BV2 microglial cells and HT22 hippocampal neurons, ordered from the American Type Culture Collection (ATCC, Rockville, MD, USA), were grown in an incubator with 5% CO_2_ at 37°C with the use of a DMEM-F12 medium supplemented with 10 mmol/L Hepes, 10% Fetal Bovine Serum (FBS, HyClone, Logan, UT, USA), and 1% Penicillin-Streptomycin Solution. The solution was replaced every two or three days, and the cells were passed every four or five days.

### *Establishment of the* in-vitro *oxygen-glucose deprivation (OGD) model*

As mentioned before [19], BV2 microglial cells and HT-22 hippocampal neurons were seeded into a glucose-free DMEM (Gibco) and then transferred to a modular incubator. They were flushed with the 3 L/min mixture of 95% N_2_ and 5% CO_2_ at room temperature for 15 minutes. After that, the chamber was sealed and put into a container at 37°C. OGD was implemented. Four hours later, the cells were moved into a normal incubator for 24 hours’ recovery, with the medium substituted by an ordinary maintenance medium. Cells in the control group were cultivated with a normal DMEM together with 10% FBS for the same time. Cells maintained in the normal medium under normal conditions were taken as controls. Twenty-four hours later, HT-22 cells were harvested for CCk8, flow cytometry, Western blot, and TUNEL staining with an aim to examine the functions of HT-22.

### Cell transfection

The pcDNA empty vector (NC, 5 μg/ml), pcDNA-CCR2 (CCR2, 5 μg/ml), the miRNA control (miR-NC, 50 nM), miR-381-3p mimics (miR-381-3p, 50 nM), the miRNA inhibitor (miR-in, 100 nM), and the miR-381-3p inhibitor (miR-381-3p-in, 100 nM) were supplied by GenePharma Co., Ltd. (Shanghai, China). OGD-elicited BV2 microglia and HT22 hippocampal neurons were seeded into 24-well cell culture plates with a density of 3 × 10^5^ cells/well. Transfection was conducted after they were cultivated in an environment of 37°C and 5% CO_2_ for 24 hours. The above-mentioned RNAs were transfected into OGD-induced BV2 microglia and HT22 neurons employing Lipofectamine® 3000 (Invitrogen; ThermoFisherScientific, Inc.). qRT-PCR was carried out to determine the efficiency of the transfection [20]. The cells were incubated under the conditions of 37°C and 5% CO_2_ for 24 hours in preparation for further analysis.

### Lactic dehydrogenase (LDH) release determination

As detailed before [21], BV2 cells induced by OGD were transfected together with miR-NC and miR-381-3p mimics for 24 hours’ further culture. Then, BV2 cells were inoculated into 96-well plates with a density of 2 × 10 ^4^cells/well. With the cell supernatant harvested, LDH-Cytotoxicity Colorimetry Kit II (#K313-500, BioVision, USA) was taken to measure LDH release in BV2 cells. Put simply, 60 μl of LDH detection solution was administered to 150 μl of the cell supernatant for incubation in darkness at 37°C. About 30 minutes later, the LDH kit was operated to gauge LDH release in the cell supernatant at 450 nm, as instructed by the supplier.

### CCK8 for cell proliferation detection

CCK8 was performed to assess cell activity. In a nutshell [22], HT-22 cells elicited by OGD were inoculated into 96-well plates with a density of 2 × 10 ^4^cells/well and then transfected along with miR-NC, miR-381-3p mimics, CCR2 overexpression plasmid, and CCR2 overexpression plasmid+miR-381-3p mimics for 24 hours’ culture. As per the instructions of the manufacturer, 10 μL of the CCK8 (Dojindo Molecular Technologies, Kumamoto, Japan) reagent was added into each hole. After that, the cells were further incubated in an incubator at 37°C for an hour. The value of OD450 was determined by a spectrophotometer (Bio-Rad, CA, USA).

### Flow cytometry (FCM)

Put simply, HT-22 cells induced by OGD, inoculated into 6-well plates with a density of 2 × 10^5^cells/well, were transfected along with miRNA, miR-381-3p mimics, CCR2 overexpression plasmid, and CCR2 overexpression plasmid+miR-381-3p mimics for 24 hours’ culture and dealt with trypsin. The collected cells followed the procedures stipulated by the apoptosis detection kit (Shanghai Aladdin Biological Reagent Co. Ltd). The cells were flushed in PBS twice, followed by the addition of 400 μL pre-cooled PBS and then 10 μL AnnexinV-FITC as well as 5 μL PI, respectively. Right after they were subsequently incubated in darkness at 4°C for 30 minutes, flow cytometry was utilized for detection. The percentage of apoptotic cells was calculated through computer software processing [23].

### Terminal-deoxynucleoitidyl transferase-mediated nick end labeling (TUNEL)

TUNEL was implemented in line with the manufacturer’s instructions with the use of TUNEL Alexa Fluor imaging assay (Invitrogen) [24]. In a word, miR-NC and miR-381-3p were transfected into OGD-induced HT22 cells which were subsequently seeded into 6 cm culture dishes equipped with cover glasses. An immunostaining fixative was taken to immobilize the cells for 30 to 60 minutes, which were rinsed in PBS once later. An immunostaining detergent was administered for 2 minutes’ incubation in an ice bath. Next, 50 μL of TUNEL detection solution was given to the samples for 60 minutes’ incubation in darkness at 37°C. PBS was utilized to rinse the samples three times. After being sealed with the anti-fluorescence quenching sealing solution, the samples were monitored under a fluorescence microscope with 450–500 nm excitation light and 515–565 nm emission light (green fluorescence). With five fields randomly chosen from each sample, the apoptotic rate was calculated as per the formula: apoptosis rate = apoptotic cells/total cells×100%.

### Enzyme linked immunosorbent assay (ELISA)

Following OGD treatment, BV2 microglia in the logarithmic growth stage, inoculated into 6-well plates, were transfected along with miRNA, miR-381-3p mimics, CCR2 overexpression plasmid, and CCR2 overexpression plasmid+miR-381-3p mimics for 24 hours’ further culture. The cell supernatant was collected and centrifuged at 1000 rpm and 4°C for 10 minutes. The supernatant after centrifugation was obtained. In the meantime, the brain tissues of HI rats and HI rats injected with miR-NC and miR-381-3p mimics via the ventricle were harvested. Then, 50–100 mg of the brain tissues in each group were administered to centrifuge tubes, followed by the addition of 0. 01 mol PBS solution as per 0. 05 g/mL. They were made into milky white suspension with the use of an ultrasonic cell disruptor. Subsequent to 10 minutes’ centrifugation at 12,000 r/min, the supernatant was harvested. Enzyme-linked immunosorbent assay (ELISA) was implemented to check the contents of IL-1β, IL-6, and TNF-α in line with the instructions of the ELISA kit (Abcam, Shanghai, China) [25,26].

### Real-time quantitative polymerase chain reaction (qRT-PCR)

As mentioned before [27], TRIzol reagent (Invitrogen™, Carlsbad, California, USA) was harnessed to extract total RNA from the cells. miRNA miR-381-3p and CCR2 were reverse-transcribed employing the TaqMan MicroRNA reverse transcription kit (Applied Biosystems, Foster City, CA, USA) and the PrimeScript™ RT Reagent kit (Invitrogen, Shanghai, China). With the first-strand cDNA obtained, qRT-PCR was carried out employing the TaqMan miRNA detection kit (Applied Biosystems), the SYBR Kit, and SYBR Green dye (MedChemExpress, NJ, USA), respectively. PCR was performed as the following: 5 minutes of pre-denaturation at 95°C; 15 seconds of denaturation at 95°C; and 30 seconds of annealing at 60°C. GAPDH was employed as the internal reference to determine the profile of CCR2, and U6 was adopted as the internal reference to detect miR-381-3p. Statistical analysis was carried out via the 2^(-ΔΔCt)^ approach. Each experiment was duplicated three times. The primers’ information is detailed in Table 1.

**Table 1.** Primer sequences for RT-PCR

Gene	Forward primers	Reverse primers
miR-381-3p	TAGATGAACCACCTGCCTCG	AGACAGGACATGGAGAGCTG
CCR2	GGGAGCCAAAAGGGTCAT	GAGTCCTTCCACGATACCAA
U6	CTCGCTTCGGCAGCACA	AACGCTTCACGAATTTGCGT
GAPDH	GGGA GCCAAAAGGGTCAT	GAGTCCTTCCACGATACCAA

### Western blot

As described before [28], BV2 and HT-22 cells, induced by OGD and transfected along with miR-NC and miR-381-3p, were flushed in PBS three times, lysed on ice using RIPA lysis buffer (Beyotime Biotcchnology, Shanghai, China) for 10 minutes, and centrifuged with a high-speed freezing centrifuge at 4°C and 14,000 g/min for 10 minutes. As the supernatant was harvested, the protein concentration was gauged through the BCA method. The HI rats’ brain tissues were collected, with 1 mL of pre-cooled tissue lysis buffer administered every 100 mg. After homogenization with an ultrasonic cell disruptor in an ice bath, the supernatant was harvested, and the protein concentration was measured through the BCA approach. Next, 50 μg of total protein was added into 12% polyacrylamide gel, followed by 2 hours’ electrophoresis at 100 V. Then, the protein was electrically moved onto polyvinylidene fluoride (PVDF) membranes (Millipore, Bedford, MA, USA). After being sealed with 5% skimmed milk powder for an hour at room temperature and flushed in TBST three times (10 minutes each), the membranes were incubated overnight at 4°C along with primary antibodies Anti-Bad (1:1000, ab59348, Abcam, MA, USA), Anti-Bax (1:1000, ab32503, Abcam), Anti-cleaved-caspase3 (1:1000, ab32042, Abcam), Anti-iNOS (1:1000, ab178945, Abcam), Anti-COX2 (1:1000, ab179800, Abcam), Anti-TLR4 (1:1000, ab13556, Abcam), Anti-CCR2 (1:1000, ab203128, Abcam), Anti-NF-κB (1:1000, ab16502, Abcam), Anti-p-NF-κB (1:1000, ab76302, Abcam), and Anti-β-actin (1:1000, ab8227, Abcam). Following TBST washing, they were incubated along with the HRP-labeled anti-rabbit secondary antibody (concentration: 1:3000) at room temperature for an hour. TBST was utilized to rinse the membranes another three times, 10 minutes each. Eventually, Western blot reagent (Invitrogen) was applied for color imaging, and the ImageJ 1.44 software was introduced for densitometry analysis.

### Dual luciferase activity assay

Both luciferase reporter vectors (CCR2-WT and CCR2-MUT) were engineered by Promega, Inc. (Promega, Madison, WI, USA). As described earlier [29], BV2 and HT-22 cells (4.5 × 10^4^) were seeded into 48-well plates and grown to 70% confluence. CCR2-WT and CCR2-MUT, along with miR-381-3p mimics or the negative control, were transfected into BV2 and HT-22 cells with the use of liposome 2000. Forty-eight hours after the transfection, the luciferase activity was examined on the basis of the supplier’s instructions. All experiments were performed in triplicate and duplicated three times.

### RNA immunoprecipitation assay (RIP)

As per the instructions of the manufacturer, RIP was conducted with the Magna RIP kit (EMD Millipore, Billerica, MA, United States) [30]. RIP lysis buffer was adopted for lysing the cells, followed by the addition of the human anti-AgO-2 antibody (Millipore) or the control antibody (normal mouse immunoglobulin, Millipore). And then the cells were incubated overnight at 4°C. qRT-PCR was implemented to check the profile of CCR2 in the lysate.

### The HI rat model

Newborn Sprague Dawley (SD) rats (1–2 days after birth) were provided by the Experimental Animal Center of Nanchang University. All the experiments on the rats were implemented in line with the National Institutes of Health guidelines for the care and use of laboratory animals. The research program had received the imprimatur from the Animal Ethics Committee of Yichun University (approval number: YCEC-2020-065). Sixty newborn SD rats were randomized to four different groups (15 rats in each group): the Sham group (N = 15), the HI group (N = 15), the HI+miR-NC group (N = 15), and the HI+miR-381-3p mimics group (N = 15). HI was operated, as previously stated[31]. Put simply, pentobarbital sodium (40 mg/kg) was given to anesthetize the rats. As for the rats in the sham group (N = 15), only their neck skin was cut open to expose the left common carotid arteries under which a suture was passed, without ligation and hypoxia treatment. In the HI group (N = 45), the animals’ neck skin was cut open following anesthesia, with their left common carotid arteries dissociated and ligated. An hour later, the animals were placed in an oxygen-deficient chamber in which the combination of 8% O_2_ and 92% N_2_ was transfused at the speed of 1 ~ 2 L/min for two hours. After the HI surgery, 15 rats in the HI group were taken out for intraventricular injection (ICV) under anesthesia. miR-381-3p mimics and the negative control miR-NC were ordered from Guangzhou Rizhao Biotechnology Co., Ltd. (Guangzhou, China). Next, 1 μL of miR-381-3p mimics or miR-NC was intraventricularly injected into the rats as per the following stereoscopic coordinates: front −0.9 mm, side 1.8 mm, and front reg −3.8 mm. Twenty-four hours after the injection, the rats were intraperitoneally transfused with 50 mg/kg pentobarbital sodium and sacrificed through cervical dislocation, with their brain tissues harvested for histopathological examination.

### Tissue immunofluorescence

The profile of CCR2 was determined through immunofluorescence [32]. Frozen slices of fresh brain tissues were immobilized with 4% formaldehyde solution for 10 minutes, permeated with Triton X. 100 for 15 minutes, and blocked with the immunostaining blocking solution. Then the specimens were incubated overnight in a refrigerator at 4°C along with the primary antibody CCR2 (1:300) and then incubated at room temperature for an hour. After that, they were dyed with 4ʹ6-diamidine-2-phenylindole (DAPI) for three to five minutes. At last, the slices were sealed and monitored under a fluorescence microscope.

### Nissl staining

As described before [33], subsequent to 24 hours’ hypoxia/ischemia, 5 rats randomly chosen from each group were intraperitoneally transfused with 50 mg/kg pentobarbital sodium and then sacrificed through cervical dislocation. Then their heads were cut off to obtain the brains, which were immobilized with 4% paraformaldehyde for 24 hours. The tissues were routinely dehydrated, made transparent, embedded, severed into coronary slices (4 m in thickness), and transformed into paraffin sections. Following all the steps, toluidine blue was added for Nissl staining. At last, a person not in the know randomly chose five non-overlapping fields in the ischemic penumbra cortex from each section to take photos with the use of a 400x microscope (Olympus, Japan).

### Immunohistochemistry assay

Immunohistochemistry was employed to examine the profile of Iba1 [34]. The fixed tissues were routinely dehydrated, embedded, and sliced up, followed by baking at 60°C for an hour. Xylene I, xylene II, and xylene III were employed respectively to dewax the sections for 5 minutes. Next, the samples were rinsed in absolute ethyl alcohol, 95% ethyl alcohol, and 75% ethyl alcohol, respectively, for five minutes, and then flushed in water for five minutes. A citrate buffer solution was utilized for 10 minutes’ microwave reparation. After cooling at room temperature, the samples were incubated without any light in a wet box for 15 minutes along with 0.3% hydrogen peroxide. The goat serum blocking solution was put in use for another 30 minutes’ incubation in the wet box at 37°C. The slices were cultured with the anti-Iba1 antibody (1:300; ab178846; Abcam, MA, USA) overnight at 4°C. With the HRP-labeled secondary antibody working solution administered, they were incubated in a wet box at 37°C, followed by coloring with diaminobenzidine (DAB). The sections were washed in water, dehydrated, made transparent, and observed. At last, the Image-Proplus software (Media Cybernetics, USA) was introduced to analyze the average gray value of the positive expression sites.

### Statistical analysis

Student t test was conducted for comparison between two groups, while ANOVA was adopted to compare multiple groups in combination with Tukey-Kramer. All outcomes were presented as mean ± SEMS. The experiment was implemented in triplicate. The GraphPad Prism software (version 8.0) was exploited for drawing. *P < 0.05* was regarded as statistically meaningful.

## Results

We first constructed an in-vitro HI model in microglia and neurons. The miR-381-3p level was detected. Next, functional assays was performed to confirm the role of miR-381-3p in microglial activation and neuronal apoptosis. The downstream mechanism of miR-381-3p was investigated.

### OGD triggered down-regulated miR-381-3p profile in microglial cells and neurons

**Figure 1.**
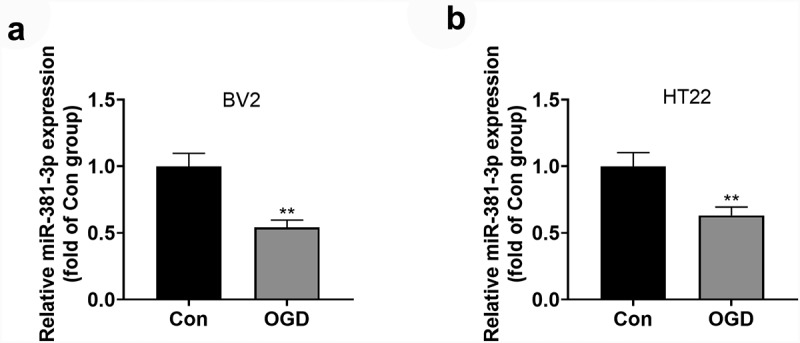
OGD triggered miR-381-3p down-regulation in the microglia and neurons. A and B: qRT-PCR determined miR-381-3p expression in BV2 microglia and HT22 neurons induced by OGD. **P < 0.01 (vs. the Con group). Statistics were presented as mean ± SD, N = 3.

To understand the expression features of miR-381-3p in the OGD-elicited *in-vitro* HI model, an OGD model was engineered in BV2 microglia and HT22 neurons. qRT-PCR ascertained miR-381-3p’s level in cells, indicating that in contrast with the normal group, miR-381-3p expression was notably leveled down in BV2 microglial cells and HT22 neurons that were influenced by OGD (Figure 1(a-b)). Those data suggested that miR-381-3p partook in OGD-mediated BV2 and HT22 cells.

### miR-381-3p overexpression attenuated inflammation in OGD-treated BV2 microglia

**Figure 2.**
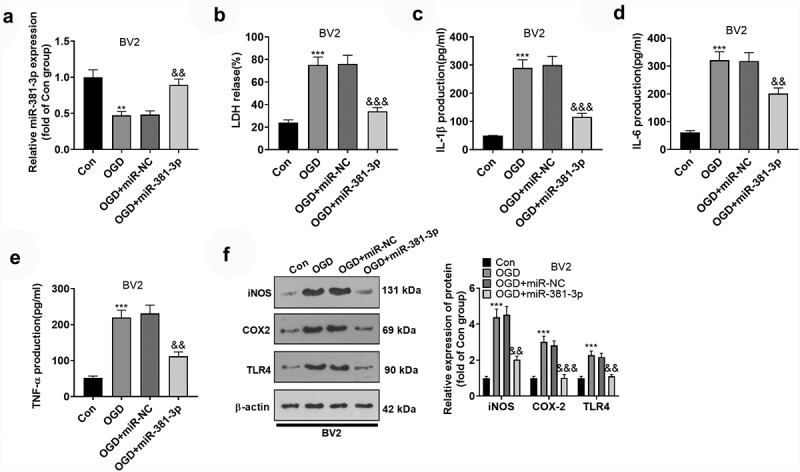
Overexpression of miR-381-3p abated inflammation in OGD-treated BV2 microglia. A. miR-381-3p mimics and the negative control (miR-NC) were transfected into BV2 microglia induced by OGD. qRT-PCR examined miR-381-3p in the microglia. B: The LDH level in BV2 microglia was determined. C-E: ELISA evaluated the profiles of IL-6, IL-1β, and TNF-α in the culture medium of OGD-induced BV2 microglia. E: Western blot ascertained the protein profiles of iNOS, COX-2, and TLR4 in OGD-elicited BV2 microglia. ** p < 0.01, ***p < 0.001 (vs. the Con group). &&p < 0.01, &&&p < 0.001 (vs. the OGD+miR-NC group). Statistics were presented as mean ± SD, N = 3.

To delve into the influence of miR-381-3p on BV2 microglial cells, we transfected miR-381-3p mimics in OGD-elicited BV2 microglia. qRT-PCR detected the up-regulation of miR-381-3p expression (Figure 2(a)). First of all, the LDH activity in BV2 microglia was figured out, which reflected that the activity of LDH was substantially stepped up following OGD. That signaled the microglia were in a situation of hypoxia, and miR-381-3p up-regulation abated the LDH activity (Figure 2(b)). To further corroborate whether heightened expression of miR-381-3p alleviated microglia-mediated neuroinflammation, we carried out ELISA and Western blot to figure out the profiles of inflammatory factors IL-6, IL-1β, and TNF-α as well as inflammatory proteins iNOS, COX-2, and TLR4. As revealed by the outcomes, by contrast to the Con group, inflammatory factors were augmented in BV2 microglia induced by OGD, the protein levels of iNOS, COX-2, and TLR4 were elevated, and miR-381-3p overexpression could lower the profiles of inflammatory cytokines and inflammatory proteins (Figure 2(c-f)). Given these results, overexpression of miR-381-3p attenuated inflammation in BV2 microglia after OGD treatment.

### Overexpression of miR-381-3p mitigated neuron damage

**Figure 3.**
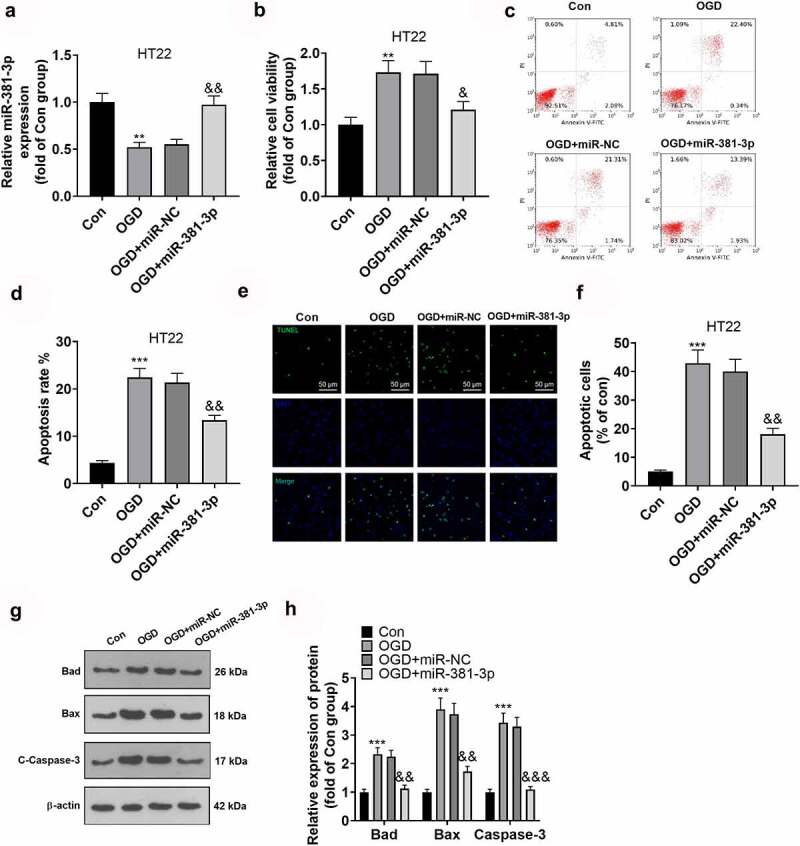
Overexpression of miR-381-3p alleviated neuronal injury. miR-381-3p mimics and the negative control (miR-NC) were transfected into HT22 hippocampal neurons under OGD treatment. A: qRT-PCR ascertained the profile of miR-381-3p in HT22 neurons. B: CCK8 assay evaluated HT22 cell viability. C-D: Flow cytometry (c) and TUNEL assay (d) tracked HT22 cell apoptosis. Scale = 50 μm. E: Western blot detected apoptotic proteins (Bad, Bax, Caspase3) in HT22 cells. **P < 0.01, ***P < 0.001 (vs. the Con group), *&P < 0.05, &&P < 0.01* (vs. the OGD+miR group). Statistics were displayed as mean ± SD, N = 3.

To dig deeper into the function of miR-381-3p in hippocampal neurons, we transfected hippocampal neurons along with miR-381-3p mimics to enhance miR-381-3p expression (Figure 3(a)). The proliferation and apoptosis of hippocampal neurons were examined through CCK8 and flow cytometry. The outcomes denoted that in contrast with the Con group, the OGD group witnessed a decline in hippocampal neurons’ proliferation and a rise in their apoptosis. Overexpression of miR-381-3p expanded the proliferation of hippocampal neurons (Figure 3(b)) and restrained neuron apoptosis (Figure 3(c)). To corroborate the outcomes of flow cytometry, we implemented TUNEL to track cell apoptosis and discovered that by contrast to the Con group, hippocampal neuron apoptosis was stepped up in the OGD group. Overexpression of miR-381-3p contributed to a decline in neuron apoptosis (Figure 3(d)). Eventually, Western blot was operated to ascertain the protein profiles of Bad, Bax, and c-Caspase-3 in HT22 neurons. In light of the results, in contrast with the Con group, the protein profiles of Bad, Bax, and Caspase-3 in the hippocampal neurons were uplifted in the OGD group but were abated after miR-381-3p was overexpressed (Figure 3(e)). Collectively, miR-381-3p overexpression reduced OGD-mediated neuron apoptosis.

### CCR2 was targeted by miR-381-3p

**Figure 4.**
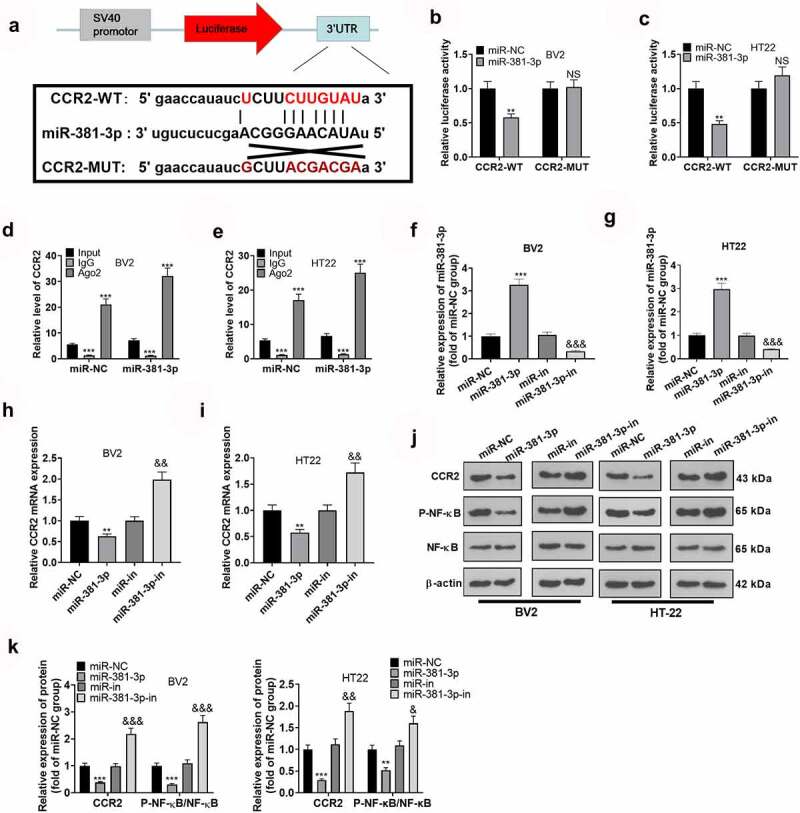
CCR2 was targeted by miR-381-3p. A: The binding site between miR-381-3p and CCR2 was forecast via ENCORA (http://starbase.sysu.edu.cn/). B: Luciferase reporter gene analysis investigated the correlation between miR-381-3p and CCR2 in BV2 and HT22 cells. C: RIP confirmed the correlation between miR-381-3p and CCR2. The enrichment of CCR2 in BV2 and HT22 cell lysates was examined by qRT-PCR. **P < 0.01, ***P < 0.001 (vs. the input group). D: The CCR2 mRNA level in BV2 and HT22 cells transfected along with miR-381-3p mimics or the miR-381-3p inhibitor was verified by qRT-PCR. E. The protein levels of CCR2 and NF-κB in BV2 and HT22 cells transfected along with miR-381-3p mimics or the miR-381-3p inhibitor were confirmed via Western blot. NSP>0.05, **P < 0.01, ***P < 0.001 (vs. the miR-NC group). *&P < 0.05, &&P < 0.01* (vs. the miR-in group). Statistics were displayed as mean ± SD, N = 3.

The above works have signified that miR-381-3p up-regulation can dampen microglial inflammation ad suppress neuron apoptosis. Nevertheless, the exact mechanism remains poorly understood. To grasp the certain mechanism of miR-381-3p, we conducted bioinformatics analysis via ENCORA (http://starbase.sysu.edu.cn/), which displayed the binding site between CCR2 3’-UTR and miR-381-3p (Figure 4(a)). Dual luciferase reporter assay revealed that miR-381-3p mimics lessened the luciferase activities of BV2 microglia and HT22 neurons transfected with CCR2-WT but had no significant effects on luciferase activities of BV2 microglia and HT22 neurons transfected with CCR2-MUT (Figure 4(b)). Next, we implemented RIP assay in BV2 microglia and HT22 neurons. As exhibited in the outcomes, the enrichment of CCR2 precipitated in the Ago2 antibody group was markedly higher than that in the IgG group. This signified that CCR2 combined with Ago2 through miR-381-3p (Figure 4(c)). In BV2 microglia and HT22 neurons induced by OGD, overexpression and down-regulation of miR-381-3p led to the down-regulation and up-regulation of CCR2 mRNA, respectively (Figure 4(d)). Moreover, Western blot reflected that miR-381-3p overexpression curbed CCR2 and NF-κB phosphorylation (Figure 4(e)). These findings pointed out that CCR2 was a functional target of miR-381-3p.

### miR-381-3p abated microglial inflammation and hippocampal neuronal apoptosis triggered by overexpression of CCR2

**Figure 5.**
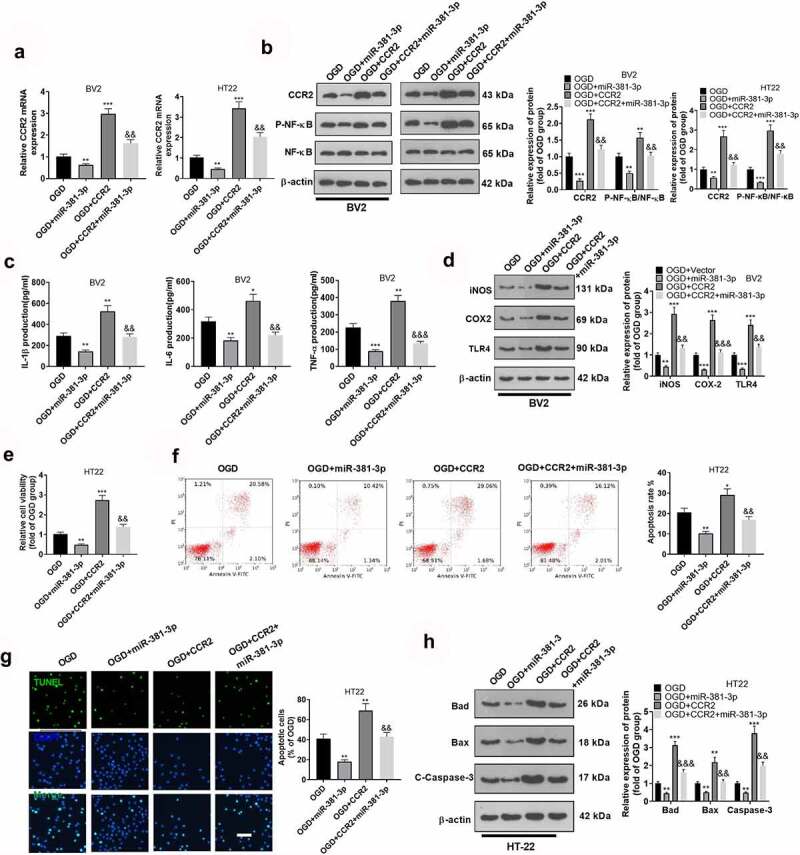
miR-381-3p weakened microglial inflammation and hippocampal neuron apoptosis resulting from overexpression of CCR2. CCR2 overexpression plasmid and miR-381-3p mimics were transfected together into BV2 microglia or HT22 neurons induced by OGD. A and B: CCR2 expression in BV2 microglia and HT22 neurons was determined by qRT-PCR and Western blot. C: ELISA was implemented to confirm the profiles of IL-6, IL-1β, and TNF-α in BV2 microglia. D: Western blot was utilized to check the profiles of inflammatory proteins (iNOS, COX-2, TLR4) in BV2 microglia. E: CCK8 monitored HT22 neuron proliferation. F: HT22 neuron apoptosis was tracked by flow cytometry. G: TUNEL assay ascertained the apoptotic level of HT22 cells. Scale = 50 μm. H: The profiles of apoptotic proteins (Bad, Bax, and Caspase3) in HT22 neurons were figured out via Western blot. *P < 0.05, **P < 0.01, ***P < 0.001 (vs. the OGD group), *&&P < 0.01, &&&P < 0.001* (vs. the OGD+CCR2 group). Statistics were displayed as mean ±SD, N = 3.

The above findings disclosed that CCR2 was targeted by miR-381-3p. To assess whether inflammation sparked by miR-381-3p was directly mediated by CCR2, we transfected OGD-elicited BV2 microglia and HT22 neurons along with CCR2 overexpression plasmid and miR-381-3p mimics. As suggested by Western blot, miR-381-3p up-regulation could abate the profile of CCR2 in BV2 microglia and HT22 hippocampal neurons as well as NF-κB phosphorylation (Figure 5(a, b)). In light of ELISA and Western blot, up-regulating CCR2 could heighten the profiles of IL-1β, IL-6, TNFα, iNOS, COX-2, and TLR4 in BV2 microglia, whereas overexpression of miR-381-3p could lower them (Figure 5(c, d)). CCK8, flow cytometry, and TUNEL staining examined the proliferation and apoptosis of HT22 neurons. The outcomes displayed that CCR2 up-regulation could lessen hippocampal neuron proliferation (Figure 5(e)) and bolster the apoptosis (figure 5(f, g)). Overexpression of miR-381-3p could weaken the functions of CCR2. At last, Western blot confirmed the protein profiles of Bad, Bax, and Caspase-3 in hippocampal neurons, pinpointing that CCR2 up-regulation elevated the protein profiles of Bad, Bax, and Caspase-3 in hippocampal neurons, whereas overexpression of miR-381-3p brought down their expressions (Figure 5(h)). These discoveries unveiled that miR-381-3p abated microglial inflammation and hippocampal neuron apoptosis resulting from overexpression of CCR2.

### miR-381-3p expression could ameliorate the injury in the CA1 region of the HI model

**Figure 6.**
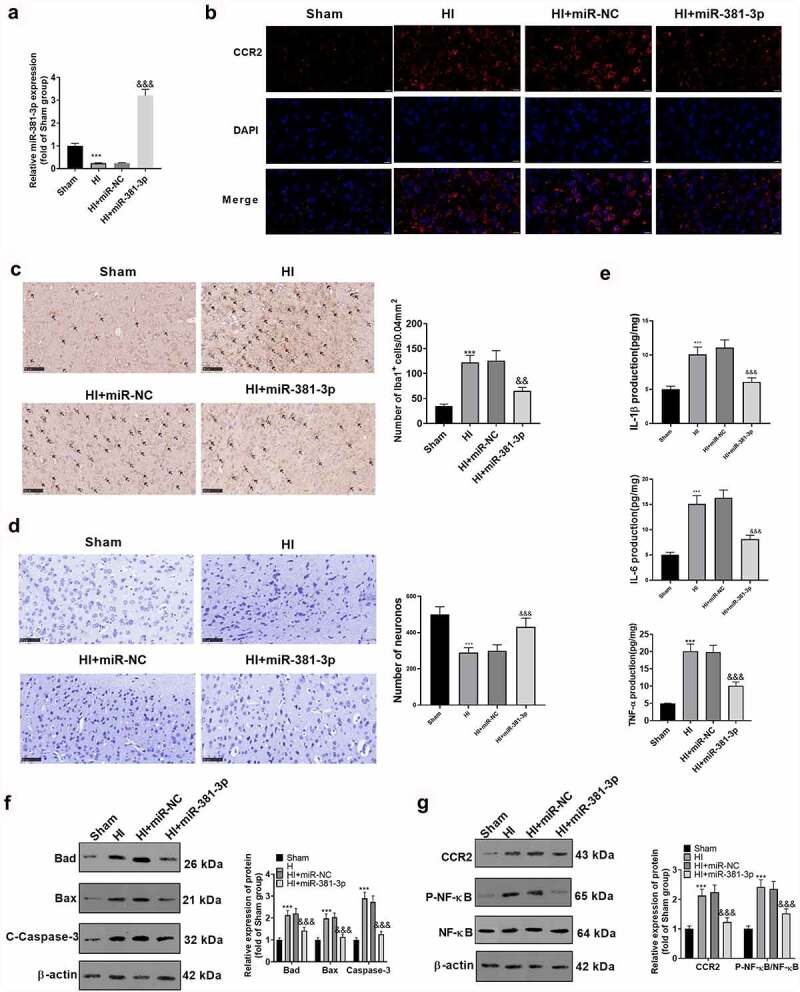
miR-381-3p overexpression ameliorated neuron apoptosis in the CA1 region of the HI rat model. An HI model was engineered in newborn rats, and they received the intracerebroventricular injection of miR-381-3p mimics or miR-NC. A: qRT-PCR confirmed miR-381-3p expression in the hippocampus of the HI rats. B: Tissue immunofluorescence checked the profile of CCR2. Scale = 50 μm. C: Immunohistochemistry verified the amount of Iba1 in the hippocampus of the HI rats. D: ELISA revealed the profiles of IL-6, IL-1β, and TNF-α in the hippocampus of the animals. E: Nissl staining was operated to verify the number of Nissl bodies in the hippocampus of the rats. F and G: Western blot was conducted to gauge the profiles of apoptotic proteins and CCR2/NF-κB in the hippocampus of the HI rats. ***P < 0.001 (vs. the Sham group), *&&P < 0.01, &&&P < 0.001* (vs. the HI+miR-NC group). Statistics were exhibited as mean ± SD, N = 5.

To validate the influence of miR-381-3p on hypoxic-ischemic encephalopathy *in vivo*, we engineered an HI rat model. First, qRT-PCR disclosed a low profile of miR-381-3p in the hippocampus of HI rats. The result displayed that miR-381-3p was lowly expressed in the hippocampus of HI rats (compared to the sham group). Notwithstanding, the injection of miR-381-3p mimics up-regulated miR-381-3p (versus the HI+miR-NC group) (Figure 6(a)). Tissue immunofluorescence was adopted to determine the profile of CCR2, indicating that in contrast with the Sham group, CCR2 expression was uplifted in the HI group. Following miR-381-3p up-regulation, CCR2s protein profile in the hippocampus of the HI rats went down (Figure 6(b)). Immunohistochemistry was employed to check the number of Iba1 cells in the hippocampus of the rats, exhibiting that miR-381-3p up-regulation lowered the number of the cells (Figure 6(c)). Through ELISA, it turned out that up-regulating miR-381-3p attenuated the profiles of IL-6, TNF-α, and IL-1β in the hippocampus of the HI rats (Figure 6(d)). The hippocampal tissues of the rats were harvested for Nissl staining to track hippocampal neuron apoptosis. In contrast with the normal group, Nissl bodies were substantially abated. Nevertheless, when the cells were transfected along with miR-381-3p, the number of the substances prominently went up (Figure 6(e)). Western blot also displayed that in contrast with the HI group, up-regulating miR-381-3p contributed to the reduction in the profiles of apoptotic proteins (figure 6(f)). Finally, Western blot was implemented to confirm the protein profile of CCR2/NF-κB. As per the results, CCR2 expression was elevated, and NF-κB phosphorylation was stepped up in the HI group. When miR-381-3p was up-regulated, the protein profile of CCR2 was lowered in the hippocampus of the HI rats, and NF-κB phosphorylation was attenuated (Figure 6(g)). It turned out that miR-381-3p restrained the CCR2/NF-κB axis, thus abating inflammation in the hippocampus of the HI rats.

## Discussion

Our work has demonstrated that the profile of miR-381-3p goes down in the *in-vivo* HI rat model and the *in-vitro* OGD model, and miR-381-3p overexpression can lessen BV2 microglia inflammation, enhance neuronal survival, and attenuate neuronal apoptosis. Additionally, the research has displayed that CCR2 may be a functional target gene of miR-381-3p. miR-381-3p may play a pivotal role in hypoxic/ischemic encephalopathy, and CCR2 may be its direct target gene. This provides guidance for basic research and potential clinical therapies in the future.

Microglia are innate immune cells of the central nervous system. Their activation is deemed to be critical in the neuroinflammatory and pathological progression of ischemic tissues[35]. Currently, it has been found that inflammation mediated by microglia exerts a prominent function in degenerative brain diseases[36], HIBD[37], and traumatic brain injury[38]. As we all know, neurons are the biggest cells with sophisticated and highly polarized morphology. The status of microglia exerts an impact on various mechanisms of neuronal communication in the brain of healthy adults. Responding to certain stimuli or neuroinflammation like Alzheimer’s disease, Parkinson’s disease[39], chronic brain underirrigation[40], and viral encephalitis[41], these cells have the capabilities of damaging and killing neurons [42,43]. Prior works have disclosed that following OGD treatment in BV2 microglia, the number of inflammatory cytokines will rise [44,45]. As a result, in this study, we have adopted OGD to treat BV2 microglia and noticed that the profiles of inflammatory factors were uplifted in the cells. Furthermore, in our study, we have analyzed neuronal injury arising from OGD from the perspectives of cell activity and apoptosis. The results denote that OHG contributes to an increase in neuron apoptosis and a decrease in neuron proliferation. From this aspect, we maintain that OGD can efficaciously trigger microglial inflammation and neuronal cell apoptosis and impede neuron proliferation resulting from OGD.

Multiple functions of miR-381-3p have been identified by previous studies. It functions as a tumor-suppressing gene by restraining tumor cell proliferation and metastasis in thyroid cancer [46] and oral squamous cell carcinoma[47]. miR-381-3p is indispensable to inflammatory diseases. For instance, in terms of LPS-triggered inflammation of acute respiratory distress syndrome (ARDS), miR-381-3p presents a low expression, and KCNQ1OT1 silencing inhibition may attenuate LPS-caused ARDS inflammation via up-regulating miR-381-30 and modulating ETS2[48]. In the high glucose-caused vascular smooth muscle cell (VSMC) dysfunction model, the profile of miR-381-3p is vigorously downgraded, and the transfection of miR-381-3p mimics can hamper inflammation and oxidative stress in VSMCs[49]. Preceding works have corroborated that miR-381-3p also has these significant functions in neuroinflammation. In MCAO rat models, miR-381-3p presents a low expression, but overexpression of miR-381-3p can hinder TNF-α signaling axis activation, facilitate EPC angiogenesis, and curb inflammation via silencing MapK38 or Cebpb, thus guarding against ischemic stroke[12]. Nevertheless, the function of miR-381-3p in hypoxic-ischemic encephalopathy (HIE) remains obscure. Therefore, we have conducted a study on it and discovered that the data we obtained are aligned with the above experimental outcomes. miR-381-3p is down-regulated in OGD-affected cells and the hippocampal tissues of HI rats. Moreover, miR-381-3p up-regulation suppresses microglial inflammation, expands neuron proliferation, and lessens neuron apoptosis. Given these results, miR-381-3p may play a positive part in hypoxic-ischemic encephalopathy.

CC motif chemokine ligand 2 (CCL2) is a chemical attractant of white blood cells like monocytes, T cells, and natural killer cells. It plays an essential role in sustaining the integrity and functions of the brain. It will give rise to inflammation through its combination with CC chemokine receptor 2 (CCR2) [50,51]. CCL2 and its receptor CCR2 are induced and take part in a multitude of neurodegenerative diseases like Alzheimer’s disease, multiple sclerosis, and ischemic brain injury[52]. As per recent studies, CCR2ʹs mRNA and protein expressions are remarkably heightened in the HIBD brain tissues of newborn rats, but its specific functions are far beyond our knowledge[53]. The transcription factor nuclear factor κB (NF-κB) family is a critical regulator of immune development, immune response, inflammation, and cancer. The NF-κB signaling system reacts to diverse stimuli. It can trigger unique cells that receive certain signals after ligand-receptor involvement[54]. The activation of NF-κB, a transcription factor driven by CCR2, exerts a critical function in dendritic cell (DC) maturation (like migration, co-stimulation, and IL-12p70 generation)[55]. On the other hand, CCL2 can elicit NF-κB signaling pathway activation via CCR2, hence exacerbating inflammation in astrocytes [56]. But how the CCR2/NF-κB signaling axis functions in hypoxic-ischemia is still unknown. Furthermore, previous research has indicated that miRNA can target the CCR2 signaling pathway to influence the inflammatory mechanism in many diseases[18]. Therefore, predicated on the above studies, we have investigated the functional mechanisms of miR-381-3p and the CCR2/NF-κB signaling pathway in hypoxic-ischemic encephalopathy. It transpires that CCR2/NF-κB presents an up-regulated profile in OGD-induced cells and HI rats’ hippocampal tissues. *In vitro*, CCR2 overexpression can strengthen NF-κB phosphorylation, step up microglial inflammation, curb neuron proliferation, and bolster neuron apoptosis, whereas overexpression of miR-381-3p can weaken the functions. Through an online website search, dual luciferase assay, and RIP, it has been disclosed that miR-381-3p can bind itself to CCR2, and these two substances are negatively correlated. These outcomes have verified that miR-381-3p can dampen microglial inflammation through curbing CCR2/NF-κB, thus ameliorating hippocampal neuron damage.

## Conclusion

To conclude, we have ascertained miR-381-3p’s functional significance in BV2 microglial cells and neurons mediated by OGD and delved into the underlying mechanism of how miR-381-3p attenuates inflammation and neuronal apoptosis. Our studies may offer us a new target to explore the mechanism of hypoxic-ischemic encephalopathy as well as a potential target to treat patients suffering from that disease.

## Supplementary Material

Supplemental Material

## Figures and Tables

**Table:** 

CCR2	C-C chemokine receptor type 2;
DMEM	Dulbecco’s Modified Eagle’s Medium;
DMSO	dimethylsulfoxide;
ELISA	Enzyme-linked immunosorbent assay;
FCM	Flow cytometry;
FBS	Fetal bovine serum;
LDH	Lactic dehydrogenase;
MTT	Momotetrazolium;
miRNA-in	miRNA inhibitor
miR-381-3p-in	MiR-381-3p inhibitor
NC	Negative control
NS	No significance
OGD	Oxygen-glucose deprivation
PBS	phosphate buffer saline
PVDF	Polyvinylidene fluoride
qRT-PCR	Quantitative polymerase chain reaction
RIP	RNA immunoprecipitation assay
SD	Sprague Dawley
TUNEL	Terminal deoxynucleotidyl-transferase-mediated 2′-deoxyuridine 5′-triphosphate nick-end labeling assay

## Data Availability

The data sets used and analyzed during the current study are available from the corresponding author on reasonable request.
