# Supplementary material for: Overexpression of microRNA-381-3p ameliorates hypoxia/ischemia-induced neuronal damage and microglial inflammation via regulating the C-C chemokine receptor type 2 /nuclear transcription factor-kappa B axis
Source: Bioengineered. 2022 Mar 4;13(3):6839–55. doi: 10.1080/21655979.2022.2038448 (PMC8973660; doi:10.1080/21655979.2022.2038448)
Supplement: Supplemental Material [file KBIE_A_2038448_SM5301.pdf]

Approval Of Ethic Committee

Date of Application 申请日期: 2020 年 06 月 25 日

The Project<br>项目名称	**Overexpression of miR-381-3p ameliorates hypoxia/ischemia-induced neuronal damage and microglial inflammation via regulating the CCR2/NF-κB axis**
Major Investigators<br>主要研究者	**Yuanmei Che, Jianglong He, Xiaopeng Li, Daxian Wu, Yi Zhang, Guicai Yuan**
Department<br>专业&科室	**Department of Infection, the Second Affiliated Hospital of Yichun University**
Approval NO.<br>伦理编号	**YCEC-2020-065**
Classification<br>研究分类	1 病理标本实验 Pathology specimens research ( )<br>2 人体实验 Human research ( )<br>3 动物组织或细胞实验 Animal tissue or cell experiments (√)<br>4 其他研究 Other research ( )
Conclusion<br>审查意见	【√ 】同意 Approved<br>【 】修正后同意 Agree after revision (Specify modification below or in accompany letter) ______<br>【 】不同意 Disagree (Specify reasons below or in accompany letter) ___
Statement<br>审查声明	兹证明本研究的设计和方法符合相关法规和伦理原则的要求。伦理委员会批准本研究项目在本院执行。<br>This is to certify that the design and methods of the research are in accordance with the requirements of related regulations and procedures as well as the ethical principles. The IRB has approved the research to be conducted in our hospital.<br>宜春学院 伦理委员会<br>宜春学院<br>Yichun University<br>2020 年 06 月 30 日
